# Dietary inclusion of black soldier fly (*Hermetia illucens*) larvae meal improves growth metrics, carcass quality, nutrient digestibility, serum analytes and cecal microbiota in Japanese quail

**DOI:** 10.1016/j.psj.2025.106119

**Published:** 2025-11-14

**Authors:** Hanan Al-Khalaifah, Ihtisham ul haq, Muhammad Tahir Khan, Muhammad Tahir, Maria Munir, Rifat Ullah Khan, Shabana Naz, Ala Abudabos, Ibrahim A. Alhidary

**Affiliations:** a Environment and Life Sciences Research Center, Kuwait Institute for Scientific Research, Safat, Kuwait; b Department of Animal Nutrition, Faculty of Animal Husbandry & Veterinary Science, The University of Agriculture, Peshawar, Pakistan; c Department of Human Nutrition, The University of Agriculture, Peshawar, Pakistan; d Physiology Lab, College of Veterinary Sciences, Department of Animal Husbandry and Veterinary Sciences, The University of Agriculture, Peshawar, Pakistan; e Department of Zoology, Government College University, Faisalabad, Pakistan; f Department of Food and Animal Sciences, College of Agriculture, Tennessee State University, Nashville, TN 37209, USA; g Department of Animal Production, College of Food and Agriculture Science, King Saud University, Riadh, Saudi Arabia

**Keywords:** Carcass traits, Feed efficiency, Growth metrics, Gut health, *Hermetia illucens*, Nutrient utilization, Serum biochemistry

## Abstract

This study evaluated the effects of replacing soybean meal with black soldier fly (*Hermetia illucens*, BSF) larvae meal on growth performance, nutrient digestibility, carcass traits, serum metabolites, and cecal microbiota of Japanese quail. A total of 240 one-day-old quail were randomly assigned to four dietary treatments: a soybean-meal control (BSF0) and diets in which BSF meal replaced soybean meal at 10 % (BSF10), 15 % (BSF15), or 20 % (BSF20) of the ration. Birds were reared for 35 days in a completely randomized design with five replicates of 12 birds each and provided feed and water ad libitum. Quail fed BSF diets showed significantly greater feed intake, final body weight, total weight gain, and average daily gain than controls (P < 0.01), with the most efficient feed conversion observed at 15 % inclusion (P < 0.001). Carcass weight, yield, and breast/thigh proportions were also superior in BSF15 (P < 0.01). Dry matter, organic matter, and crude protein digestibility were highest in BSF15 birds (P < 0.01), while ether extract digestibility peaked in BSF10 (P < 0.001). Serum total protein, cholesterol, HDL, and LDL were unaffected, but triglycerides declined in all BSF groups (P < 0.05). Cecal counts of the tested bacteria were unchanged (P > 0.05). Feed efficiency was optimal at 15% inclusion. These findings indicate that partially defatted Black Soldier Fly (BSF) meal can replace up to 15% of dietary soybean meal in quail diets to improve growth performance, nutrient utilization, and carcass yield without adverse effects on blood metabolites or gut microbiota.

## Introduction

The poultry industry faces pressure to find sustainable, cost-effective feed ingredients that reduce dependence on conventional proteins (Rahman et al., 2017; Abedinia et al., 2020; Magnoli et al., 2024; Al-Suwailem et al., 2024;Dinasarki et al., 2024; Habib et al., 2024). Japanese quail (*Coturnix japonica*) are attractive for their rapid growth, early maturity, high reproductive rate, and efficient feed conversion (Al-Khalaifa et al., 2025). Their lean, high-protein meat appeals to health-conscious consumers (Sallam et al., 2023), but feed accounts for about 70 % of production costs, prompting a search for alternative proteins (Abedinia et al., 2017; Amiri et al., 2024).

Soybean meal is widely used for its amino acid balance and digestibility (Hafeez et al., 2021; Boonmee et al., 2024) but is limited by high price, market volatility, and concerns about genetically modified crops (Aini et al., 2023; Ciptaan et al., 2024). Fish meal faces similar cost and sustainability issues (Hall, 2010). Insects, especially black soldier fly (BSF; *Hermetia illucens*) larvae meal, are a promising substitute (Lu et al., 2022). BSF larvae convert organic waste into biomass with 40–60 % crude protein and 20–35 % fat (Tariq et al., 2025), supply balanced amino acids and bioavailable minerals, and contain medium-chain fatty acids such as lauric acid with antimicrobial and immunomodulatory effects (Franco et al., 2025). Their production supports circular bioeconomy goals (Pratama et al., 2024; Camperio et al., 2025). Poultry studies show BSF improves growth and nutrient use without harming health (Hayat et al., 2024), enhances gut function and meat quality (Fiorilla et al., 2024), and modulates cecal microbiota and short-chain fatty acids in laying hens.

In quail, BSF meal improved growth and meat quality, boosted performance and microbiome balance when fed whole (Liu et al., 2025), enhanced meat characteristics (Rahmawati et al., 2021), and supported immunity (Harlystiarini et al., 2020). Its digestible protein and lipids aid nutrient use, while antimicrobial peptides and lauric acid reduce *E. coli* and *Salmonella* and support *Lactobacillus* spp. (Meng et al., 2023). Antioxidant and serum-lipid benefits have also been noted (Dabbou et al., 2021). Optimal inclusion rates remain uncertain. Moderate levels (10–15 %) often improve performance, but higher amounts may lower efficiency due to indigestible chitin or excess fat (Meng et al., 2023; Nguyen et al., 2023). This study tested partially defatted BSF larvae meal at 10, 15, and 20 % as a soybean meal replacement in Japanese quail diets, evaluating growth, carcass traits, nutrient digestibility, serum biochemistry, and cecal microbiota to define practical inclusion levels for sustainable production.

## Materials and methods

### Experimental birds and management

**Fig. 1:**
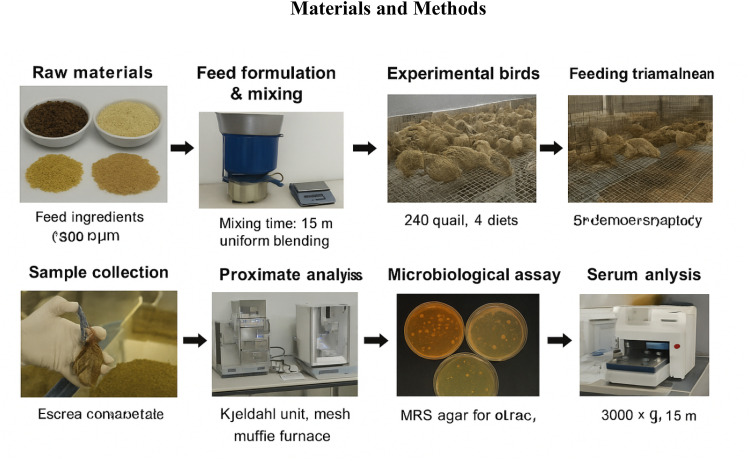
Sequential workflow of animal preparation, diet formulation, and sample analysis in Japanese quailsfed black soldier fly (*Hermetia illucenus*) larvae meal.

A total of 240 one-day-old Japanese quail (*Coturnix japonica*) were obtained from a local supplier and raised under uniform husbandry conditions. Birds were allotted to four experimental diets in a completely randomized design, with 60 chicks per treatment and five replicates of 12 birds each. The feeding trial lasted 35 days, including one week of adaptation, during which feed and water were available without restriction. Quail were housed in floor pens bedded with wood shavings and kept under continuous 24-h illumination. Ambient temperature was maintained between 33 and 35°C during the first week and gradually reduced to 24–26°C by the end of the experiment, with relative humidity ranging from 55 to 65 %. To maintain flock health, rigorous biosecurity measures were applied, including routine disinfection of facilities, controlled entry to the premises, and strict sanitation practices. Sequential workflow of animal preparation, diet formulation, and sample analysis in Japanese quailsfed black soldier fly (*Hermetia illucenus*) larvae meal is shown in Fig. 1.

### Experimental diets

Four experimental diets were prepared: Control (basal diet with soybean meal); BSF10 (10 % black soldier fly (*Hermetia illucens*) larvae meal); BSF15 (15 % BSF meal); and BSF20 (20 % BSF meal). Diets were formulated for starter (0–3 wk) and finisher (4–6 wk) phases (Table 1). Black soldier fly meal (proximate composition in Table 2) was partially defatted, finely ground (<500 µm) and thoroughly incorporated. All rations were isonitrogenous and isoenergetic, meeting or exceeding nutrient requirements.

**Table 1:** Ingredients composition and proximate analysis of the experimental diet.

Ingredient (%)	Starter				Finisher diet			
	Control	BSF_10_	BSF_15_	BSF_20_	Control	BSF_10_	BSF_15_	BSF_20_
Wheat grains	20	20	20	20	20	20	20	20
Corn grains	37	35	35	37	40	40	40	40
Soybean Meal	20	15	10	0	20	15	10	0
BSF Meal	0	10	15	20	0	10	15	20
Wheat Bran	10	10	10	10	8	4	4	8
Soya Oil	6	3	3	6	5	4	4	5
Limestone	1.5	1.5	1.5	1.5	1.5	1.5	1.5	1.5
Dicalcium Phosphates	1.5	1.5	1.5	1.5	1.5	1.5	1.5	1.5
Additives	1	1	1	1	1	1	1	1
DL-Methionine	0.2	0.2	0.2	0.2	0.2	0.2	0.2	0.2
Mineral Premix	1	1	1	1	1	1	1	1
salts	0.8	0.8	0.8	0.8	0.8	0.8	0.8	0.8
Trace mineral	1	1	1	1	1	1	1	1
Total	100	100	100	100	100	100	100	100
**Proximate Analysis**								
DM	88.1	88.4	89.5	87.9	86.1	86.5	87.5	87.9
CP	25	25.7	25.3	25.2	25	25.7	25.3	25.2
EE	5.34	6.74	8.52	9.83	5.34	6.74	8.52	9.83
Ash	8.74	9.25	8.04	7.76	3.64	4.59	5.27	5.99

BSF_0_; basal diet, BSF_10_; basal diet with 10 % Black Soldier Fly larvae meal, BSF_15_; basal diet with 15 % Black Soldier Fly larvae meal, BSF_20_; basal diet with 20 % Black Soldier Fly larvae meal, DM; dry matter, CP; crude protein, EE; ether extract.

**Table 2:** Proximate analysis of Black soldier fly larvae meal.

Nutrient	Composition (g/kg)
DM	180.23 ± 2.11
CP	561.44 ± 4.36
EE	19.23 ± 1.08
CF	13.26 ± 0.92
Ash	9.2 ± 0.53
NDF	397.31 ± 3.42

DM; dry matter, CP; crude protein, EE; ether extract; CF, crude fiber; NFE, Nitrogen free Extract.

All feed ingredients and chemicals used in formulation and proximate analysis were of analytical grade. Chromic oxide (Cr₂O₃; CAS No. 1308-38-9; ≥99 % purity; Sigma-Aldrich, USA), petroleum ether (CAS No. 8032-32-4; ≥99.5 %; Merck, Germany), sulfuric acid (CAS No. 7664-93-9; 95–98 %; Merck, Germany), sodium hydroxide (CAS No. 1310-73-2; ≥98 %; Sigma-Aldrich, USA), and boric acid (CAS No. 10043-35-3; ≥99.5 %; Merck, Germany) were used in the proximate and digestibility analyses.

### Proximate methodology for BSF larvae

The proximate composition of BSF larvae meal was determined using standard AOAC (2000) procedures to quantify moisture (oven drying at 105°C), crude protein (Kjeldahl method, *N* × 6.25), crude fat (Soxhlet extraction with petroleum ether), crude fiber (acid–base digestion), ash (muffle furnace incineration at 550°C), and nitrogen-free extract (calculated by difference). Prior to analysis, BSF larvae were oven-dried, finely ground, and partially defatted by Soxhlet extraction with petroleum ether to reduce excess lipid and ensure a uniform nutrient profile suitable for dietary incorporation.

### Growth performance

Growth metrics were recorded over the 35-day feeding trial. Initial body weight (IW) of individual quail was measured on day 1, and final body weight (FW) was taken at the end of the experiment using a calibrated digital balance (AND GF-3000, Japan; ±0.1 g). Pen-based daily feed intake (FI, g bird⁻¹ day⁻¹) was determined gravimetrically by subtracting feed refusals from the amount offered. Total weight gain (TWG, g bird⁻¹) was calculated as TWG = FW – IW, and average daily gain (ADG, g bird⁻¹ day⁻¹) was derived from TWG/35. Feed conversion ratio (FCR) was expressed as the ratio of total FI to TWG, with adjustments for mortality by including the body weight of any dead birds in the calculations. Mortality was checked each morning and recorded to correct intake data.

### Carcass evaluation

At the conclusion of the trial, birds were fasted for 12 h, individually weighed to obtain slaughter weight (SW), and then humanely slaughtered. Carcasses were eviscerated and weighed to determine carcass weight (CSW) and carcass yield (CSP, % of SW). The combined weight of thighs and breast (WTB) and its proportion relative to SW (PTB) were also measured.

### Apparent nutrient digestibility

A digestibility assay was performed during the final week of the 35-day trial to evaluate the effects of dietary BSF larvae meal on nutrient utilization. Four quail from each treatment (one bird per replicate) were randomly chosen and housed individually in wire-mesh metabolic cages (45 × 30 × 30 cm; stainless steel, locally fabricated) designed for total excreta collection, preventing contamination of droppings with feed particles. Birds underwent a 2-day adaptation period in the cages, followed by a 4-day quantitative collection phase. Feed and water were offered ad libitum, and total feed intake was recorded for each bird. Fresh excreta were collected twice daily (morning and evening), pooled per bird, weighed, oven-dried at 60°C for 72 h, finely ground, and stored in airtight containers pending analysis. The chemical composition of both the experimental diets and excreta was determined according to Ullah et al. (2022) procedures to obtain values for dry matter (DM), organic matter (OM), crude protein (CP), and ether extract (EE). Apparent digestibility coefficients were calculated as:

$$\text{Apparent}\;\text{digestibility}=100-\left[100\;\times \frac{\text{Nutrient}\;\left(\%\right)\;\text{in}\;\text{digesta}\times \text{Cr}\left(\%\right)\text{in}\;\text{feed}}{\text{Nutrient}\left(\%\right)\text{in}\;\text{feed}\times \text{Cr}\left(\%\right)\text{in}\;\text{digesta}}\right]$$

Where Cr = chromic oxide (CAS No. 1308-38-9).

### Serum biochemistry

Blood samples were collected from the brachial vein prior to slaughter. Serum was separated by centrifugation (Eppendorf 5810R, Germany; 3,000 × *g* for 15 min at 4°C) and analyzed for total protein, albumin, globulin, cholesterol, triglycerides, high-density lipoprotein (HDL), and low-density lipoprotein (LDL) using commercial diagnostic kits (Randox Laboratories Ltd., UK; analytical range as per manufacturer’s protocol).

### Cecal microbiota assessment

On the final day of the experiment (day 42), two birds from each replicate were randomly selected and humanely euthanized by cervical dislocation in compliance with institutional animal-care protocols. Cecal digesta were aseptically collected for microbial enumeration. Approximately 1 g of cecal content from each bird was homogenized in buffered peptone water (BPW; CAS No. 7558-79-4; HiMedia Laboratories, China) at a 1:10 ratio and spread onto selective agar media specific to the target organisms. *Lactobacillus* spp. were cultured on de Man–Rogosa–Sharpe (MRS) agar (HiMedia Laboratories, Shingi, Chna; Cat. No. M641) under 5 % CO₂ at 37°C for 24 h. *Salmonella* spp. and *Escherichia coli* were plated on eosin methylene blue (EMB) agar (Hardy Diagnostics, Santa Maria, CA, USA) and incubated at 37°C for 24 h. All microbial cultures were performed in duplicate, and the detection limit for viable counts was 10² CFU g⁻¹ (equivalent to 2.0 log₁₀ CFU g⁻¹). Colony counts were performed with a digital colony counter and expressed as log₁₀ colony-forming units per gram of digesta (log₁₀ CFU g⁻¹).

### Statistical analysis

Data were analyzed using one-way ANOVA in a completely randomized design. Means were compared using Tukey’s multiple range test, and significance was declared at *P* < 0.05. Results are presented as mean ± standard error of the mean (SEM). All statistical analyses were performed using SPSS software (version 26.0, IBM Corp., Armonk, NY, USA).

## Results

The influence of graded levels of BSF larvae meal on the growth responses of Japanese quail is presented in Table 3. Although the initial body weight of the chicks was statistically similar across all treatments (*P* > 0.05), birds receiving BSF-based diets displayed a clear improvement in subsequent performance indices. Feed intake increased progressively as the proportion of BSF meal in the diet rose, and this was accompanied by significant elevations in final body weight, total weight gain, and average daily gain compared with the control group (*P* < 0.01 to *P* < 0.001). Among the experimental treatments, the diet containing 15 % BSF meal (BSF15) produced the most favorable feed conversion ratio, which was significantly better than both the control diet and the highest inclusion level (BSF20) (*P* < 0.001), indicating that moderate inclusion promoted the most efficient utilization of nutrients for growth.

**Table 3:** Effect of dietary inclusion of various levels of black soldier fly larvae meal on the growth performance of quails.

Diets	IW (g)	Feed intake (g/bird)	FW (g/bird)	TWG (g/bird)	ADG (g/bird)	FCR
BSF_0_	9.8	12.81^d^	132.2^d^	122.4^d^	2.8^d^	4.40^a^
BSF_10_	10.2	13.22^c^	155.6^c^	145.4^c^	3.4^c^	3.82^c^
BSF_15_	9.2	14.23^b^	176.2^a^	166.9^a^	3.9^a^	3.58^d^
BSF_20_	10.3	15.88^a^	163.5^b^	153.1^b^	3.8^b^	4.32^b^
SEM	0.02	0.04	0.28	0.6	0.04	0.06
P-value	NS	**	**	**	***	***

Means in the same column with different superscripts (^abcd^) are significantly different (*P* < 0.05). NS; non-significant, **p < 0.01; ***p < 0.05; BSF_0_; basal diet, BSF_10_; basal diet with 10 % Black Soldier Fly larvae meal, BSF_15_; basal diet with 15 % Black Soldier Fly larvae meal, BSF_20_; basal diet with 20 % Black Soldier Fly larvae meal, IW; Initial weight, FE; Final weight, ADG; average daily gain.

Carcass characteristics followed a pattern that mirrored the live-weight responses and are summarized in Table 4. Birds fed the BSF15 diet achieved the greatest slaughter weight, eviscerated carcass weight, and carcass yield percentage, as well as the highest absolute and relative weights of the combined thigh and breast portions (*P* < 0.01). Quail offered the BSF20 diet exhibited intermediate values that remained significantly higher than those of the control group for most carcass parameters, confirming that inclusion of BSF meal at or above 15 % supports superior carcass deposition compared with a soybean-meal–based diet.

**Table 4:** Effect of dietary inclusion of various levels of black soldier fly larvae meal on carcass traits and yield in quails.

Diets	SW (g)	CSW (g)	CSP (%)	WTB (g)	PTB (%)
BSF_0_	127.57^d^	91.81^d^	64.27^b^	34.33^d^	37.39^b^
BSF_10_	141.54^c^	96.65^c^	67.66^b^	37.47^c^	38.77^a^
BSF_15_	160.17^a^	105.74^a^	74.02^a^	42.71^a^	40.39^a^
BSF_20_	152.11^b^	100.21^b^	69.72^c^	39.24^c^	39.16^c^
SEM	0.36	0.46	0.20	0.27	0.39
P-value	***	**	**	***	***

Means in the same column with different superscripts (^abcd^) are significantly different (*P* < 0.05). **p < 0.01; ***p < 0.05; BSF_0_; basal diet, BSF_10_; basal diet with 10 % Black Soldier Fly larvae meal, BSF_15_; basal diet with 15 % Black Soldier Fly larvae meal, BSF_20_; basal diet with 20 % Black Soldier Fly larvae meal, SW; slaughter weight, CSW; carcass weight; CSP; carcass percentage; WTB; weight of thighs and breasts; PTB; percentage of thighs and breasts.

As shown in Table 5, the incorporation of BSF meal produced notable enhancements in apparent nutrient digestibility. Dry matter and organic matter digestibility coefficients rose significantly with increasing BSF levels, reaching their highest values in birds fed the BSF15 diet (*P* < 0.01). Crude protein digestibility was likewise superior in BSF15 compared with both the control and the 20 % inclusion group (*P* < 0.01). Interestingly, ether extract digestibility peaked in quail receiving the BSF10 diet and was significantly greater than in all other treatments (*P* < 0.001), suggesting that moderate inclusion of BSF meal optimizes lipid utilization.

**Table 5:** Effect of dietary inclusion of various levels of black soldier fly larvae meal on nutrient digestibility in quails.

Digestibility (%)	DMD	OMD	CPD	EED
BSF_0_	57.21^d^	61.86^c^	25.33^b^	3.36^d^
BSF_10_	59.53^c^	63.42^b^	25.18^b^	8.56^a^
BSF_15_	63.53^a^	67.31^a^	26.74^a^	4.41^c^
BSF_20_	61.50^b^	62.27^c^	24.39^b^	6.37^b^
SEM	0.408	0.33	0.327	0.051
P-value	**	***	**	***

Means in the same column with different superscripts (^abcd^) are significantly different (*P* < 0.05). **p < 0.01; ***p < 0.05; BSF_0_; basal diet, BSF_10_; basal diet with 10 % Black Soldier Fly larvae meal, BSF_15_; basal diet with 15 % Black Soldier Fly larvae meal, BSF_20_; basal diet with 20 % Black Soldier Fly larvae meal, DMD; dry matter digestibility, OMD; organic matter digestibility CPD; crude protein digestibility, EED; ether extract digestibility,.

Data summarized in Table 6 reveal that dietary BSF supplementation did not alter serum total protein, albumin, globulin, albumin-to-globulin ratio, cholesterol, or the concentrations of high- and low-density lipoproteins (*P* > 0.05). Nevertheless, a significant reduction in serum triglycerides was detected in all BSF-fed groups relative to the control birds, with the most pronounced decrease observed in the BSF15 treatment (*P* < 0.05), indicating a beneficial effect of BSF meal on circulating lipid fractions. Triglyceride concentrations decreased from 98.3 mg/dL in the control group to 84.2 mg/dL in the 15 % BSF inclusion group, representing an approximate 14.3 % reduction. Other inclusion levels (10 % and 20 %) showed intermediate reductions of 7.9 % and 9.8 %, respectively, relative to the control (*P* = 0.05).

**Table 6:** Effect of diets containing graded levels of black soldier fly larvae on serum biochemical parameters of Japanese quail.

Parameter	Total Protein (g/dL)	Albumin (g/dL)	Globulin (g/dL)	Albumin/Globulin Ratio	Cholesterol (mg/dL)	Triglycerides (mg/dL)	HDL (mg/dL)	LDL (mg/dL)
BSF_0_	4.97	2.1	3.06	0.98	152.8	98.3^a^	51.8	95.8
BSF_10_	4.86	2.33	3.11	1.02	153.8	90.5^b^	52.1	95.9
BSF_15_	5.08	2.49	3.41	1.07	153.2	84.2^d^	52.4	96.2
BSF_20_	5.13	2.22	3.03	1.02	153.6	88.7^c^	52.9	96.1
SEM	0.19	0.08	0.11	0.04	2.90	2.95	1.10	1.36
P-value	0.18	0.21	0.12	0.09	0.13	0.05	0.08	0.10

Means in the same column with different superscripts (^abcd^) are significantly different (*P* < 0.05). ***p < 0.05; BSF_0_; basal diet, BSF_10_; basal diet with 10 % Black Soldier Fly larvae meal, BSF_15_; basal diet with 15 % Black Soldier Fly larvae meal, BSF_20_; basal diet with 20 % Black Soldier Fly larvae meal,.

The counts of key cecal bacteria are presented in Table 7. Neither *E. coli* nor *Salmonella* spp. nor *Lactobacillus* spp. populations were significantly affected by the inclusion of BSF meal at any dietary level (*P* > 0.05). These findings suggest that, under the conditions of the present study, moderate to high incorporation of BSF larvae meal does not disrupt the core cultivable microbiota of the quail cecum.

**Table 7:** Influence of black soldier fly larvae meal inclusion on cecal microbial count (CFU log_10_/g) in quails.

Diets	Escherichia coli	Salmonella	Lactobacillus spp
BSF_0_	4.18 ± 0.02	3.12 ± 0.03	4.34 ± 0.04
BSF_10_	4.33 ± 0.03	3.18± 0.01	4.28± 0.03
BSF_15_	4.45 ± 0.03	3.19± 0.04	4.32± 0.01
BSF_20_	4.65± 0.04	3.13± 0.04	4.39± 0.02
P-value	0.13	0.94	0.75

Means in the same column with different superscripts (^abcd^) are significantly different (*P* < 0.05). **p < 0.01; BSF_0_; basal diet, BSF_10_; basal diet with 10 % Black Soldier Fly larvae meal, BSF_15_; basal diet with 15 % Black Soldier Fly larvae meal, BSF_20_; basal diet with 20 % Black Soldier Fly larvae meal.

## Discussion

The present study demonstrated that partial substitution of soybean meal with BSF larvae meal improved growth performance, nutrient digestibility, and carcass traits in Japanese quail, with the 15 % inclusion level (BSF15) providing the most consistent benefits. These outcomes agree with previous poultry research showing that moderate dietary BSF levels (≈5–15 %) can enhance weight gain and feed efficiency compared with conventional protein sources (Schiavone et al., 2022; Dabbou et al., 2025). The greater feed intake and higher final body weight of BSF-fed birds likely reflect both the high nutrient density of BSF meal and its acceptable palatability. Defatted BSF meal provides a balanced amino acid profile and readily available energy (Dabbou et al., 2025), factors that can stimulate voluntary feed consumption and promote growth. Similar improvements in body weight and feed efficiency after partial replacement of soybean or fish meal with BSF larvae have been documented in quail (Schiavone et al., 2022). The improved growth and feed conversion ratio (FCR) can be mechanistically linked to the high lauric acid content of BSF, which enhances lipid metabolism and may reduce gut microbial load, improving nutrient assimilation (Spranghers et al., 2018; Li et al., 2021). The enhanced slaughter weight, carcass yield, and breast/thigh weights observed at 15 % inclusion parallel the growth improvements and suggest more efficient protein deposition. Comparable increases in carcass yield with moderate BSF substitution have been reported in broiler chickens and quail (Schiavone et al., 2022; Dabbou et al., 2025).

In the current study, apparent digestibility of dry matter, organic matter, and crude protein peaked at 15 % BSF inclusion. BSF meal is rich in highly digestible protein fractions and essential amino acids (Józefiak et al., 2024). Additionally, the moderate chitin content in BSF meal may stimulate endogenous chitinase secretion in the gut, enhancing nutrient breakdown and assimilation at moderate inclusion levels, while excessive chitin at higher inclusion rates (20 %) can hinder enzyme access, explaining the plateau or slight decline in digestibility (Finke, 2013; Firman et al., 2020). In addition, the moderate chitin content of insect meals may stimulate endogenous chitinase secretion and beneficial gut adaptations, improving nutrient breakdown; however, excessive chitin at higher inclusion rates can encapsulate nutrients and reduce enzyme access (Finke, 2013). This explains the plateau or slight decline in digestibility at 20 % inclusion, consistent with other poultry trials (Firman et al., 2020).

In the present study, serum triglycerides declined significantly in BSF-fed birds, whereas cholesterol, HDL, and LDL were unchanged. BSF larvae contain substantial medium-chain fatty acids, particularly lauric acid, which are rapidly oxidized for energy rather than stored as triglycerides (Spranghers et al., 2018). Lauric acid’s hypolipidemic effect is mechanistically linked to enhanced medium-chain fatty acid oxidation in the liver, promoting triglyceride clearance and improving lipid metabolism (Li et al., 2021). Additionally, the mild antimicrobial properties of lauric acid may influence hepatic lipid regulation. Lauric acid has also been associated with improved lipid metabolism and mild antimicrobial activity that can influence hepatic lipid regulation (Li et al., 2021).

Despite the presence of antimicrobial peptides, chitin, and medium-chain fatty acids in BSF meal, no significant differences were detected in *E. coli, Salmonella* spp., or *Lactobacillus* spp in the current study. counts. Similar neutral effects have been reported in other poultry studies, where BSF feeding altered microbial metabolites such as short-chain fatty acids without major changes in culturable bacterial counts (De Marco et al., 2015). To further explore gut microbial responses, future studies should employ DNA-based approaches such as 16S rRNA sequencing to assess microbial diversity beyond culture-dependent methods. Differences in BSF processing and larval substrate likely influence the concentration of bioactive compounds and thus microbial responses (Józefiak et al., 2024).

The superior performance at 15 % and the less favorable feed conversion at 20 % highlight an optimal inclusion window. Higher BSF levels may increase indigestible chitin and residual fat, reducing nutrient availability and gut efficiency (Barragan-Fonseca et al., 2017). Our study did not measure intestinal histomorphology, short-chain fatty acids, or detailed microbial community structure, which could provide mechanistic insight into the observed effects. Our work did not measure intestinal histomorphology, short-chain fatty acids, or detailed microbial community structure, which could clarify underlying mechanisms. Furthermore, BSF nutrient profiles vary with rearing substrate; reporting proximate and fatty-acid composition is essential for comparison across studies.

## Conclusion

Inclusion of partially defatted Black Soldier Fly (*Hermetia illucens*) larvae meal at approximately 15 % of the diet optimizes growth performance, nutrient digestibility, and carcass yield in Japanese quail, while maintaining normal serum lipid profiles and stable cecal microbiota. These findings support BSF meal as a sustainable and effective alternative to soybean meal in quail nutrition.

## Funding

Not applicable.

## Ethical approval

This experiment was reviewed and approved by the Ethical Committee of the Department of Poultry Science, Faculty of Animal Husbandry and Veterinary Sciences, The University of Agriculture, Peshawar, Pakistan (Notification No. l-452/AH/UAP, dated 16 November 2022).

## CRediT authorship contribution statement

**Hanan Al-Khalaifah:** Funding acquisition. **Ihtisham ul haq:** Methodology, Investigation, Data curation. **Muhammad Tahir Khan:** Supervision, Resources, Project administration, Conceptualization. **Muhammad Tahir:** Supervision. **Maria Munir:** Formal analysis, Data curation. **Rifat Ullah Khan:** Validation, Software. **Shabana Naz:** Writing – review & editing, Writing – original draft. **Ala Abudabos:** Writing – review & editing, Writing – original draft. **Ibrahim A. Alhidary:** Writing – review & editing, Writing – original draft.

## Disclosures

Authors declare no conflict of interest
